# A responsible authorship culture is needed and it is a collective responsibility

**DOI:** 10.1371/journal.pbio.3003745

**Published:** 2026-03-26

**Authors:** Véronique Kiermer, Kirsten Bibbins-Domingo, Magdalena Skipper

**Affiliations:** 1 Public Library of Science, San Francisco, California, United States of America; 2 Journal of the American Medical Association and the Journal of the American Medical Association Network, Chicago, Illinois, United States of America; 3 Nature, London, United Kingdom

## Abstract

In this Formal Comment, representatives from PLOS, Nature and JAMA call for action on adopting a principle-based approach for a responsible authorship culture.

Questionable authorship practices have long been a concern for scientific integrity because they create a climate of mistrust in researchers and their findings. Deliberately hiding the contributions of some and naming undeserving individuals as authors both threaten research integrity.

Another dimension of this problem is that research standards evolve and when authorship practices remain stagnant, important contributions may go unrecognized. For example, researchers who perform critical tasks to ensure the development and integrity of data, code, and methods, are increasingly important. Yet an authorship tradition of rewarding only “intellectual contributions” often leads to critical contributors being left off the author list. Also, important contributors may be based outside academia but the processes by which authorship decisions are made may not always include them.

We participated in a working group convened by the US National Academies of Sciences, Engineering, and Medicine, where our perspectives as journal editors were complemented by those of researchers at various career stages, authorship and integrity scholars, and universities representatives. The group concluded that for a more responsible authorship culture to embed and endure, authorship decisions must be anchored in three interconnected principles: credit, accountability and transparency [1].

The group examined the public authorship guidelines of a range of US research institutions and found room for improvement. But this problem is by no means limited to US institutions. In our experience, the issues are global, and we call on institutions worldwide to address this critical issue and lead in the establishment of a better authorship culture by inculcating and supporting good practice.

Institutions are essential to establishing and reinforcing authorship standards and have a unique opportunity through the incentives and disincentives they control that influence the practice of authors. But authorship standards and culture is an individual and collective responsibility. The working group’s analysis also showed that journals’ guidance is variable and sometimes inadequate to support the situations emerging in modern research. While journals cannot adjudicate authorship practice, they can provide better guidance. We commit our journals to this process and call on journals and the author community to engage in helping set these standards.

The credit-accountability-transparency framework and the concrete recommendations published by the working group are tools that give us all an opportunity to revisit how we can support a fair and responsible authorship culture anchored in credit, accountability and transparency.

This piece is simultaneously published in JAMA, PLOS Biology and PLOS Medicine. An abbreviated version appeared as a Correspondence in Nature.
